# Gunshot Wound of the Abdomen, with Perforation of the Intestines

**Published:** 1887-08

**Authors:** Frank T. Andrews


### GUNSHOT WOUND OF THE ABDOMEN, WITH PERFORATION OF THE INTESTINES.

BY FRANK T. ANDREWS, A. M., M. D.

THE following case is of interest in showing how the varying conditions in actual practice frequently interfere with the performance of laparotomy for gunshot wounds of the abdomen.

A gentlemen, fifty-seven years of age, of good physique and temperate habits, received a bullet-wound of the abdomen from a thirty-two caliber revolver.

After receiving the injury, the patient walked at least a quarter of a mile, and rode several miles in a buggy over country roads. He was then brought to this city, a distance of sixty miles, by rail.

When I first saw the patient, at least eight hours after the accident, he was suffering from overwhelming shock and from internal hæmorrhage. The extremities were cold. The temperature in the axilla 96.4° F. The pulse was nearly imperceptible at the wrist, and at intervals, when it could be counted, it was one hundred and fifty per minute.

In marked contrast to the radial pulsations, the contractions of the heart were strong. The apex beat was visible to the eye and perceptible to the touch.

Auscultation revealed a diastolic murmur over the aortic valves, indicating insufficiency and consequent regurgitation.

The heart, equal to its task before the accident, was unable properly to perform its functions under the new conditions.

The question, of course, arose whether there was any reasonable prospect that the patient could survive a laparotomy for cleansing the peritoneal cavity and closing the wounds of the intestines. It was evident to me that, in his moribund condition, the operation would only hasten the fatal termination. It was therefore not performed.

The patient died in about thirty hours without reacting from the shock.

The post-mortem examination, as made by the county physician, showed that the bullet, entering the body an inch and a half to the left of the median line, at the lower border of the ninth rib, passed almost directly downward through the anterior edge of the diaphragm, perforated the small intestine in two places, passed through the ala of the ilium and lodged behind the great trochanter.

There was considerable clotted blood, but no fecal matter in the peritoneal cavity. The peritoneum was not inflamed.

Possibly an operation, immediately after the injury and before the patient was weakened by traveling and by the loss of blood, would have been successful.

